# Relationship between the Thickness of the Coracoid Process and Latarjet Graft Positioning—An Anatomical Study on 70 Embalmed Scapulae

**DOI:** 10.3390/jcm9010207

**Published:** 2020-01-12

**Authors:** Markus Gregori, Lukas Eichelberger, Claudia Gahleitner, Stefan Hajdu, Michael Pretterklieber

**Affiliations:** 1 Department of Orthopedics and Trauma-Surgery, Vienna General Hospital, Medical University of Vienna, Währinger Gürtel 18-20, 1090 Vienna, Austria; 2 Department of Orthopedics and Trauma-Surgery, Krankenhaus der Barmherzigen Brüder Eisenstadt, Johannes von Gott-Platz 1, 7000 Eisenstadt, Austria; 3 Center for Anatomy and Cell Biology, Division of Anatomy, Medical University of Vienna, Währinger Straße 13, 1090 Vienna, Austria; michael.pretterklieber@meduniwien.ac.at

**Keywords:** shoulder, instability, Latarjet, glenoid, bone defect, graft position

## Abstract

Background: The Latarjet procedure is a popular technique with the aim of the reconstruction of glenoid cavity bone defects in patients with chronic anterior shoulder instability. Studies have shown that the Congruent arc Latarjet procedure is better able to reconstruct larger defects than the Classic Latarjet, but there is a lack of information on the limitations of both methods. Methods: The dimensions of the glenoid width and the native coracoid process of two groups with 35 Formol-Carbol embalmed scapulae each were measured using a digital caliper. The relationship between the coracoid graft and the anterior-posterior diameter of the glenoid cavity was calculated to determine the maximum defect size of the glenoid cavity width, which can be treated by both Latarjet techniques. Results: The average restorable defect size of the anterior segment of the glenoid cavity was 28.4% ± 4.6% (range 19.2%–38.8%) in the Classic Latarjet group, and 45.6% ± 5.2% (range 35.7%–57.1%) in the Congruent arc Latarjet group. Based on our results, the feasibility of the Classic Latarjet procedure to reconstitute the anatomical width of the glenoid cavity was 86% in a 25% bone loss scenario, and only 40% in a 30% bone loss scenario. Conclusion: Based on our results we are unable to define a clear threshold for the optimal Latarjet graft position. In glenoid cavity defects <20%, the Classic Latarjet technique usually provides enough bone stock for anatomical reconstruction. Defects ≥35% of the glenoid cavity width should only be treated with a coracoid graft in the Congruent arc position. In the critical area between 20% and 35% of bone loss, we suggest the preoperative assessment of coracoid dimensions, based on which the graft position can be planned to restore the anatomical anterior-posterior diameter of the glenoid cavity.

## 1. Introduction

In patients with recurrent shoulder instability, the incidence of bone lesions of the anterior and anterior-inferior parts of the glenoid cavity is high—in some studies up to 70%–90% of the cases [1,2,3]. Studies have revealed that the rate of recurrent shoulder dislocations remains high, if patients with quantitative bone loss of at least 20%–25% are treated with soft-tissue repair alone [4,5]. More recently, unacceptable outcomes following arthroscopic Bankart reconstruction have led to a new definition of subcritical bone loss, if the glenoid cavity defect size exceeds 13.5% of its original width [6].

The threshold which indicates the necessity for glenoid bone augmentation is not clearly defined. Most authors recommend reconstruction of the glenoid bone stock if the amount of the anterior glenoid defect exceeds 20%–25% [4,7,8,9].

Current concepts of anterior glenoid bone augmentation involve the transfer of the coracoid process, the use of tricortical iliac crest grafts and allografts [10,11,12,13]. In contrast to other bone block techniques, the Latarjet procedure shows stabilizing capsular and muscular effects additionally to the bone effect, which was best described by Patte as the so-called “triple effect” [4,14,15,16,17]. Large studies show excellent clinical results and low recurrence rates of instability between 0% and 8%. [4,18,19,20,21,22,23] However there are also studies reporting redislocations and subluxations following the Bristow–Latarjet procedure in up to 25.8% of the cases [24].

Most clinical trials describe a flush position of the coracoid bone block, lying with its under-surface attached to the scapular neck, so that it can be deemed as the current “Classic Latarjet position” [18,19,22,25,26,27]. De Beer modified the graft fixation technique to the “Congruent arc-Latarjet” [28,29], where the graft is rotated 90° externally to the scapular plane, so that the under-surface lies in line with the glenoid cavity and becomes the articular side of the graft. Studies have shown that the shape of the under-surface more accurately correlates with the articular shape of the glenoid cavity and that the trimming of the graft in a Congruent arc fashion is not necessary [11,30]. On the other hand, the Classic Latarjet position is associated with a greater bone-to-bone attachment area, which may enhance fusion rates and counteract bending forces [30,31].

Radiographic studies have proved, that the Congruent arc Latarjet is able to restore greater defects of the glenoid cavity compared with the Classic Latarjet transfer [11]. However, the influence of preparation and decortication on graft sizes is underestimated. There is only a paucity of studies investigating the two modifications of graft positions while considering bone loss during graft preparation [31,32], but it remains unclear if a graft in lying position can, in general, restore the anatomical width of the glenoid cavity in a patient with large defects (i.e., >25%). Some authors believe that surgeons do not have to consider coracoid graft dimensions in the preoperative setting in defects between 25% and 30% [32]. It has been noted that 30% of bone loss would probably be the amount where most surgeons would decide between either a Classic or a Congruent arc position [30].

Therefore, the purpose of our study was to detect a cut-off value for maximal glenoid bone loss, which should help surgeons to decide if a transfer of the coracoid process for anatomical reconstruction of the glenoid width is possible in the Classic or Congruent arc manner, or if other bone augmentation techniques should be considered. Our hypothesis was that glenoid cavity defects up to 25% can usually be treated with Classic Latarjet procedures.

## 2. Material and Methods

The study protocol was approved by the local Ethics Committee. We included 70 unpaired Formol-Carbol embalmed scapulae, of which 48 were male and 22 female. We examined 39 left and 31 right scapulae. The shapes of the glenoid cavities were pear-like throughout and the maximum width was usually located near the 3 o’clock position or slightly lower. The scapulae were allocated into two groups of 35 bones each for Classic Latarjet and Congruent arc Latarjet procedures. Prior to graft harvesting, we assessed the maximum glenoid width (GCW), the maximum length (CPL), the maximum width (CPW) and the maximum thickness (CPT) of the coracoid process along the vertical axis.

**Figure 1:**
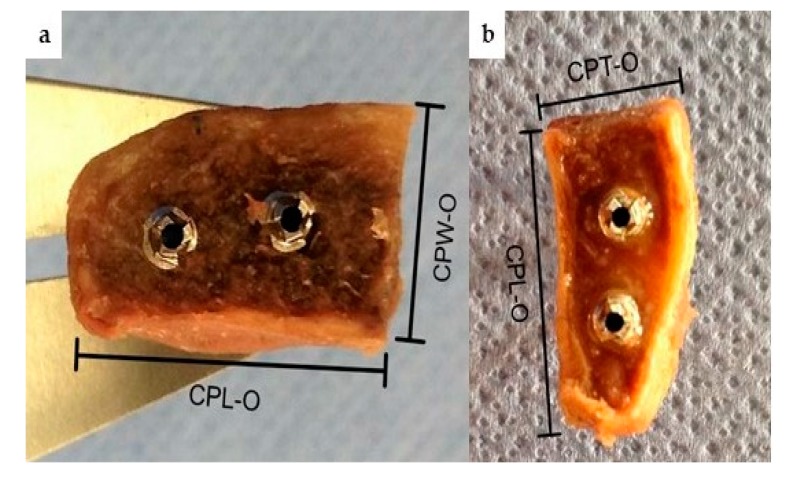
(**a**) Classic Latarjet graft after decortication and preparation. View on the under-surface of the graft showing the attachment side to the scapular neck. (**b**) Congruent arc Latarjet graft after decortication and preparation. View on the medial side of the graft showing the attachment side to the scapular neck. Maximum length (CPL-O), maximum width (CPW-O) maximum thickness (CPT-O).

**Figure 2:**
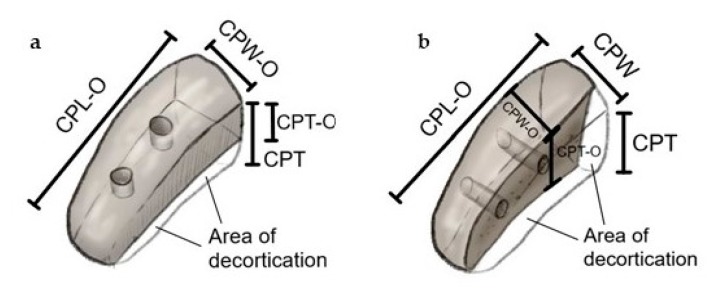
Schematic illustration of a right coracoid process for Classic Latarjet preparation (**a**) and a right coracoid process for Congruent arc Latarjet preparation (**b**). The uncolored area shows the area of decortication at the under-surface in Classic Latarjet grafts (**a**) and at the medial side of Congruent arc Latarjet grafts (**b**). All measurements prior to osteotomy were taken at intact scapulae (native thickness (CPT) and maximum width (CPW) in Figure 2a,b).

Osteotomy of the coracoid process was performed directly distal its knee with an oscillating saw (Johnson & Johnson, DePuy Synthes, Vienna, Austria) using a 10 × 0.5 mm sagittal saw blade. The bone blocks were then fixed in the bench vice in a lying position for a Classic Latarjet procedure to prepare the under-surface of the graft, and in a 90 degrees rotated position to prepare the medial border of the graft for Congruent arc Latarjet grafts. Decortication and ablation of extremely thin layers (1 mm) was performed to gain a flat cancellous bone bed, and to an amount to ensure secure and correct screw placement for potential graft fixation. (Figure 1) In Congruent Latarjet grafts, a minimal distance of 2–3 mm between the drill holes and the graft cortex was considered as secure. After the preparation of the requested cancellous area, two cannulated short-threaded cancellous screws (Ø 3.5 mm, Johnson & Johnson, DePuy Synthes, Vienna, Austria) were placed to ensure enough distance from the graft cortex. After osteotomy we assessed the maximum length (CPL-O), maximum width (CPW-O) maximum thickness (CPT-O) of the grafts. All measurements were taken using a digital caliper which displays measurements with an accuracy of 0.01 mm. Measurements were performed by one trauma surgeon, who specializes in shoulder surgery (MG), and one trauma surgeon who has worked extensively in the field of human bone and joint dissection (LE). Documentation and measurements were performed blind by each investigator. The graft dimensions were set in relation to the maximum width of the glenoid cavity to assess the maximum defect size, similar to a method already published [1]. Figure 2 demonstrates the area of decortication and measurement points.

Statistical analysis: Interobserver agreement of individual measurements of both investigators was calculated for each parameter using the intraclass-correlation-coefficient (ICC) as shown in Table 1. The Pearson correlation coefficient (Pearson’s r) was used to describe the correlation between the maximum width of the glenoid cavity (GCW) and the native thickness (CPT) or width (CPW) of the coracoid process. Data are represented as mean ± standard deviation in mm. The equality of variances for variables of both groups was calculated with the Levene test. To ensure normal distribution of the relative defect size data, we used the Shapiro–Wilk test. As the assumptions were fulfilled, a student’s *t*-test was used to compare the maximum relative defect size between the Congruent arc and the Classic Latarjet group. *p*-values less than 0.05 were considered significant. All statistical analyses were performed using SPSS, version 24, IBM Corp., 2016.

## 3. Results

At intact scapulae of both groups (*n* = 70), the mean width of the glenoid cavity was 30.1 ± 3.1 mm (median 29.6; range 25.1–38.3). The mean length, width and thickness of the coracoid process were 45.8 ± 4.4 mm (median 45.5; range 35.5–55.5), 16.8 ± 2.1 mm (median 16.3; range 13.1–22.3) and 11.7 ± 1.8 mm (median 11.3; range 8.6–15.9), respectively.

**Figure 3:**
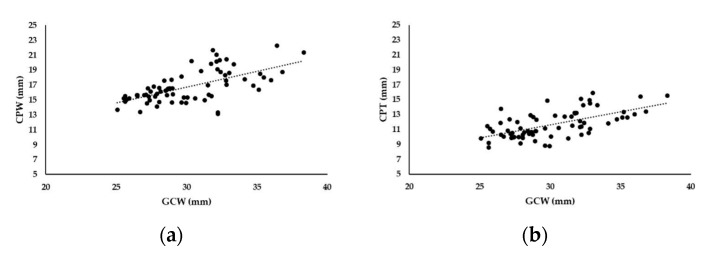
Correlation between the glenoid cavity width (GCW) and the width of the coracoid process (CPW) (**a**). Correlation between the glenoid cavity width (GCW) and the native thickness of the coracoid process (CPT) (**b**).

There was a strong correlation between the maximum width of the glenoid cavity and the width of the coracoid process (Pearson’s *r* = +0.628, *p* < 0.001) on the one hand, and between the width of the glenoid cavity and the thickness of the coracoid process (Pearson’s *r* = +0.616, *p* < 0.001) on the other (Figure 3).

Mean values for the maximum glenoid width (GCW), the native coracoid process on an intact scapula (CPL, CPW, CPT) and the coracoid process following osteotomy and preparation for graft placement (CPL-O, CPW-O, CPT-O) of both groups are shown in Table 2. In the Classic Latarjet group (*n* = 35), the mean coracoid thickness (8.4 ± 1.6 mm) was smaller compared with the Congruent arc Latarjet group (11.3 ± 1.8 mm). In contrast, the width of the Congruent arc Latarjet grafts (*n* = 35) were smaller (13.9 ± 2.2 mm) compared with those of the Classic Latarjet grafts (16.0 ± 2.0 mm).

Evaluation of the maximal glenoid defect size for Classic Latarjet procedure: After decortication of the coracoid grafts for Classic Latarjet positioning, the coracoid graft thickness declined by 3.1 ± 1.3 mm. The average relative glenoid defects size was 28.4% ± 4.6% and was calculated with the formula CPT-O*100/GCW. If we assume a 30% bone loss scenario, reconstruction of the anatomical glenoid cavity would have been possible in only 14 cases (40%), whereas 30 grafts were large enough to restore a 25% bone loss scenario (86%). The amount of maximum glenoid defect size, which could be restored by a Classic Latarjet graft ranged between 19.2% and 38.8%.

Evaluation of the maximum glenoid defect size for the Congruent arc Latarjet procedure: Following the preparation for graft fixation in a Congruent arc manner, the coracoid width declined by 3.2 ± 1.6 mm. The average relative defect size of the glenoid cavity was 45.6% ± 5.2%, which was calculated with the formula CPW-O *100/GCW. The amount of maximum glenoid defect size which could be restored by a Congruent arc Latarjet graft ranged between 35.7% and 57.1%.

**Figure 4:**
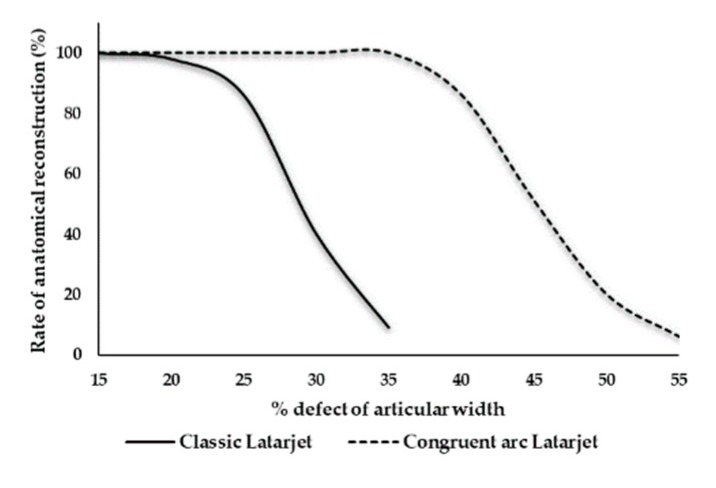
Relation between the anatomical reconstruction and defect size of the glenoid cavity for Classic Latarjet grafts and Congruent arc Latarjet grafts.

Congruent arc Latarjet grafts were able to restore a larger defect size compared with Classic Latarjet grafts (*p* < 0.001) (Figure 4).

## 4. Discussion

With this study, we wanted to detect a cut-off level regarding the maximum glenoid defect size, which can be restored by both the Congruent arc and Classic Latarjet technique, considering changes in graft dimension following the preparation and decortication of harvested bone blocks. But the main scientific value of this trial is the evidence that graft dimensions influence the Latarjet graft positioning in different bone loss scenarios. Although there was a strong correlation between dimensions of the native coracoid process and the glenoid cavity width (Figure 3a,b), the maximum restorable defect size for the anatomical reconstruction of the glenoid cavity widely ranged between 19.2% and 38.8% if a graft was used in a Classic Latarjet configuration. In a 25% bone loss scenario, anatomical reconstruction could be performed in only 86% of the cases. But already 50% of the grafts were improperly sized for defects measuring 28% of the articular width. The larger the amount of the glenoid defect, the lesser the chance that anatomical reconstruction can be achieved. Therefore, this study disproved our hypothesis that the critical threshold value for the Classic Latarjet technique is 25% of the original glenoid cavity width.

Current publications do not provide enough information on the maximal amount of defects in the anterior segment of the glenoid cavity which can adequately be restored by either the Classic or Congruent arc Latarjet procedure. The amount of bone harvest has already been published in several studies [33,34]. Of course, measurements frequently differ by a few mm. However, our results for native coracoid process dimensions do not significantly differ from those published by several authors [11,34,35]. Bueno et al. [34] proved a strong correlation between coracoid thickness and glenoid width. Our results do not significantly differ from their study, but we would have expected a more accurate correlation. However, the authors supposed that the Classic Latarjet may be limited to smaller glenoid defects less than 30% of the glenoid width. Armitage et al. [11] measured coracoid dimensions at 3D-CT-reconstructions. They have shown that a Congruent arc Latarjet is able to reconstitute greater defects than the Classic Latarjet, but they did not take into consideration bone loss during graft preparation.

Although several authors have demonstrated that bone defects of 25%–30% can be successfully treated with Latarjet grafts in lying position [20,32], it is not clear if an anatomic reconstruction of the original glenoid cavity width can be achieved in every patient as a rule. Changes of the glenoid shape correlate with the amount of bone loss [36]. If the native pear shape of the glenoid cavity becomes inverted, it usually indicates anterior bone loss of 25% [36]. In larger anterior defects, Burkart described a more banana-like shape of the glenoid [4]. It still remains unclear if a Classic Latarjet position is adequate in cases with such large defects, or if a defect between 25%–30% would necessitate a Congruent arc Latarjet or other bone augmentation techniques instead.

Beyond contention, abrasion of the attachment site of the coracoid graft is necessary to enhance bone healing [22,25,37]. Hantes et al. [32] demonstrated that the Latarjet procedure with the graft in lying position sufficiently restores the anatomy of the glenoid cavity in a model based on anatomic specimens with a 25% anterior-inferior bone defect. The mean defect area was 28.7% ± 6% of the intact glenoid. Most anatomic studies create anterior-inferior bone defects, but clinical findings revealed that bone loss of the glenoid cavity is usually located more anteriorly [38,39]. However, our results do not confirm those of Hantes et al., as we proved, that only 86% of the Classic Latarjet grafts are able to reconstruct bone defects of 25%. In our study, the average defect size in the Classic Latarjet group was 28.4% ± 4.6%, which is equivalent to the mean defect area of this study group treated with Classic Latarjet. Nonetheless, based on our results, we cannot agree that there is no need to calculate the surface area of the coracoid, because almost full reconstruction can be expected in 25%–30% scenarios [32]. According to our results, only 40% of the Classic grafts were able to reconstruct 30% of the glenoid width.

Bathia et al. [12] noted that not all their coracoid grafts were thick enough to fill up a 30% bone defect. In their study, 30% bone loss was represented by a defect width of 9.2 mm ± 0.75 mm, which correlates closely to our results, where 30% bone loss was equivalent to a defect width of 9.0 mm ± 0.9 mm. Provencher et al. noted that a 5% increment of the glenoid width comprises 1–2 mm of bone. [10] This corresponds to our findings, where 5% of the glenoid width is equal to 1.5 mm of bone.

Ghodagra et al. [40] found that in a 30% bone loss model, Latarjet grafts in a Congruent arc fashion fully restored the anatomical width, whereas grafts placed flush with their undersurface lying on the scapular neck left an average defect of 5%.

Based on our results we are unable to define a clear threshold for the optimal Latarjet graft position. Our study shows that the probability of a Classic Latarjet procedure to reconstitute the anatomical glenoid cavity width ranges between 19.2% and 38.8%. Therefore, we would consider preoperative measurements of the coracoid process in addition to the glenoid cavity defect size in the preoperative setting. The determination of both dimensions should help surgeons to assess if reconstruction of a defect larger than 20%–25% can be achieved with the graft in lying position, or if a 90° rotated position would be more suitable. Some studies show that postoperative recurrence of instability occurred in patients where the graft healed in anatomic position without any signs of bone resorption [20]. We think that more precise planning in the preoperative setting can help surgeons to decide on the best fitting graft position and prevent recurrent instability. In the Congruent arc Latarjet group, the range of anatomical reconstruction of a defect glenoid was between 35.7% and 57.1%, suggesting that large defects exceeding 30% of the articular width—which are, of course, not very common—can be successfully treated with a Congruent arc Latarjet. Despite these anatomical findings, we want to point out the important biomechanical results of Montgomery et al. [31], who showed that Classic graft fixation is stiffer than a Congruent arc graft. Additionally, they found that the attachment area is larger than in Congruent arc grafts, which may influence postoperative healing rates. Therefore, we agree with other study groups [30,31] and conclude that the graft position should be decided on a case-to-case-basis, after consideration of all the pros and cons, with the important ancillary suggestion to determine both the amount of glenoid bone loss and coracoid dimensions in the preoperative planning stage to ensure the full anatomical reconstruction of the glenoid cavity width. This seems to become important in defects involving more than 20% of the glenoid width, as our results show that the feasibility of anatomical reconstruction of the glenoid width using Classic Latarjet grafts declines from 86% in a 25% bone loss scenario, to 40% in a 30% bone loss scenario. Of course, large defects are rarely seen. Anyway, a study has shown that patients with an inverted pear shape of the glenoid cavity had a mean loss of width of 36% (25%–45%) [3].

There are several limitations in our study. Firstly, it is an anatomical investigation, which can never exactly replicate intraoperative realities. Secondly, we randomly assigned the scapulae into the two groups. At the end we had slightly more female scapulae in the Classic group (13/22) compared with the Congruent arc group (9/26). However, the dimensions of the coracoid process and the glenoid cavity width were similar in both groups. Thirdly, the sling effect provides additional stability, which has not been incorporated in this anatomic study. However, our emphasis was to find a threshold for a more accurate anatomic bony reconstruction of the glenoid width, which may influence residual instability in terms of subluxations or recurrent dislocations following Latarjet procedures. The strength of this study is that there was a strong positive correlation of the interrater measurements concerning all relevant data. Furthermore, we included a larger number of scapulae, and considered changes of graft dimensions after preparation.

## 5. Conclusions

We conclude that defects within the anterior segment of the glenoid cavity of at least 35% should be generally treated with Congruent arc Latarjet or other bone augmentation techniques. Defects lower than 20% of the glenoid width could be treated with the Classic Latarjet procedure without considering the potential amount of coracoid harvest in the preoperative setting. If bone loss of the glenoid cavity makes up between 20% and 35% of the original width, we would decide on the coracoid graft position based upon the dimensions of the coracoid process or, generally, use the Congruent arc Latarjet.

## Figures and Tables

**Table 1:** Analysis of interobserver reliability.

Measure	ICC *	*p*-Value
GCW	0.907	<0.001
CPL	0.866	<0.001
CPW	0.659	<0.001
CPT	0.910	<0.001
CPL-O	0.953	<0.001
CPW-O	0.927	<0.001
CPT-O	0.943	<0.001

* ICC, intraclass coefficient. For all other abbreviations refer to Figure 1 and Figure 2 and to the main text above.

**Table 2:** Dimensions of glenoid cavity and coracoid process prior and post graft preparation in mm (*n* = 70).

Measure	Classic Latarjet (*n* = 35)					Congruent arc Latarjet (*n* = 35)				
	Mean	Med ^⸷^	SD *	Min ^+^	Max °	Mean	Med ^⸷^	SD *	Min ^+^	Max °
GCW	29.8	29.6	3.1	25.1	36.4	30.5	29.6	3.2	25.9	38.3
CPL	44.5	44.4	4.0	35.5	52.3	47.1	46.4	4.4	38.8	55.5
CPW	16.4	16.1	2.3	13.1	22.3	17.1	16.5	2.0	14.1	21.7
CPT	11.5	11.1	1.8	8.6	15.4	11.8	11.3	1.8	8.8	15.9
CPL-O	22.0	22.1	2.6	15.8	26.3	23.0	23.6	2.5	16.6	27.4
CPW-O	16.0	15.4	2.0	12.9	21.3	13.9	13.6	2.2	10.4	18.2
CPT-O	8.4	8.3	1.6	5.8	12.7	11.3	10.9	1.8	8.3	17.0

^⸷^ Med, Median; * SD, standard deviation; ^+^ Min, minimum; ° Max, maximum.
